# Multidimensional Healthy Adult Scale: Development and validation of a measurement tool to understand how the Healthy Adult works in a Turkish population

**DOI:** 10.1371/journal.pone.0343996

**Published:** 2026-03-09

**Authors:** Duygu Yakın, Eva Billen, Raoul Grasman, Arnoud Arntz

**Affiliations:** 1 Department of Clinical Psychology, Faculty of Social and Behavioural Sciences, University of Amsterdam, Amsterdam, The Netherlands; 2 Department of Psychological Methods, Faculty of Social and Behavioural Sciences, University of Amsterdam, Amsterdam, The Netherlands; The John Paul II Catholic University of Lublin, POLAND

## Abstract

Healthy Adult (HA), a key schema therapy construct, represents the individual’s ‘healthy’ state, characterized by balancing personal and others’ needs within a realistic perspective. We developed the Multidimensional Healthy Adult Scale to explore how various dimensions of the HA contribute to different aspects of well-being and tested its factor structure and psychometric properties. Data were collected from 472 participants (24.1% male, 75.5% female) between the ages of 18 and 60. The items of the scale were generated based on a qualitative study conducted in Türkiye. Data were analyzed using Confirmatory Factor Analysis and Structural Equation Modeling in Lavaan, which demonstrated a strong fit for the measurement and predictive models. The Bond, Balance, and Battle factors, along with the overarching HA, showed good fit. Low Balance scores were associated with higher psychopathology and negative affect, while high Battle scores were associated with greater life satisfaction and positive affect. Although Bond correlated positively with Balance and Battle, high Bond scores, when controlling for the others, were linked to increased psychopathology and negative affect. These results provide evidence for a multidimensional structure of the HA. Further validation of the scale and clarification of Bond’s role is needed for clinical insights.

## Introduction

Schema therapy has been developed as treatment of chronic and persistent psychopathological states [1,2] and it is proven effective for a variety of problems [3–5] including personality disorders [6–8]. Young [1] postulated that when universal childhood needs (viz., secure attachment, autonomy, expressing needs and emotions freely, spontaneity and play, and realistic limit setting) are not met adequately, early maladaptive schemas (EMSs) develop. EMSs are pervasive patterns of making sense of the outer world that consist of cognitions, memories, and emotions [1,9]. Accordingly, limited reparenting is mostly built upon ‘correcting’ early maladaptive experiences that led to the development of EMSs. The ultimate goal of schema therapy is to reshape the internal world of the patient, enabling them to take care of their own unmet needs in their ongoing relationships with others [1]. This process begins with the therapist attuning to the needs of the patient by taking on the role of a parent within the borders of the therapeutic relationship. During therapy, patients gradually learn to take care of their own needs, helped by the modeling by the therapist. This mechanism is known as self-reparenting and considered as an important treatment goal in schema therapy, as it helps individuals to nurture and care for themselves [1,2].

According to the schema therapy perspective, when an EMS is activated in adulthood, people experience “the drama from their childhood, usually with a parent” [2] (p.10). The ‘drama’ is displayed by a group of so-called schema modes, defined as emotional states that stem from early maladaptive schemas, and the way the person copes with the schema. For example, the Vulnerable Child mode (VC) is an intense emotional state characterized by fear, abandonment and sadness that disrupts the individual’s functioning when it is triggered [1]. One mechanism of change in schema therapy is getting people out of dysfunctional schema modes by the activation of another state, the Healthy Adult mode (HA) [10]. This mode refers to the ‘healthy’ state of the person, which involves creating a balance between personal needs and the needs of others within a realistic perspective and taking responsibility for maintaining this balance. In this way, the HA plays a central role in the process of self-reparenting. The HA is defined within schema therapy through three key aspects: The HA (1) takes care of the needs of the VC by providing secure attachment and nurturance, (2) limits sudden outbursts of anger or other impulsive behavior that disturbs healthy functioning, (3) is to become responsible for fighting against the critical and demanding voices that stem from early maladaptive experiences [2].

The central techniques in schema therapy, such as empathic confrontation, chair work, imagery rescripting, and limited reparenting, stimulate the development of a sufficiently functioning HA [11,12] The central role of the HA in the change process of schema theory is emphasized both theoretically [2,9,13] and empirically [10,14]. However, what mechanisms underlie the actions of the HA in achieving mental health is not yet fully understood. To enhance the understanding of the HA as a multidimensional construct, a recent qualitative study conceptualized its three fundamental functions, as originally described in schema therapy [2] into the domains of Bond, Balance, and Battle [15]. Accordingly, each of the functions are also defined in a top-down (i.e., showing compassion, setting boundaries, and self-empowerment) as well as a bottom up (i.e., emotional attunement, self-reflection and hope and faith) levels to conceptualize how the HA functions and contributes to different assets of well-being. In addition to assessing Balance, which reflects self-reflection and the ability to set limits, Bond and Battle were designed to measure the HA’s capacity for interpersonal connection and caregiving, as well as its ability to remain activated and hopeful in the face of adversity. These factors are closely linked to empathy, compassion for others, and resilience, all of which are widely recognized in both theory and practice. However, they are not directly captured by existing measures of the HA and have therefore not been empirically tested. Given the complex nature of the self-reparenting, it is essential that this complexity is reflected in the HA and that it can be measured with precision.

In addition to the Schema Mode inventory [16], positive schemas (i.e., YPSQ [17]) and positive parenting (i.e., PPSI, [18]) can be used to measure the presence of the HA in an indirect way. The aim of this paper is not to create yet another scale for assessing the HA but to develop an instrument that captures its various dimensions, emphasizing its multidimensional conceptualization and the functions of Bond, Balance and Battle. This will hopefully enable us to understand whether these dimensions differ and contribute to changes in healthy functioning, particularly in terms of acquiring different elements of mental well-being. Thus, this paper is built upon the hypothesis that the HA is multidimensional and different dimensions operates at different levels of well-being.

Testing schema therapy constructs and measures is identified as a research priority by Pilkington et al. [19] Although measuring instruments in schema therapy have been translated and validated in several cultures, they have been developed mainly based on data from populations in the West, characterized as WEIRD (Western, Educated, Industrialized, Rich, and Democratic) [20]. In Western cultures, the conceptualization of healthy functioning places more emphasis on autonomy than on connectedness [21]. Therefore, it is crucial to ensure the applicability and presence of the HA and/or possible new dimensions that can be related to the HA in different cultures. Therefore, we aim to create an assessment tool based on qualitative data collected form a non-WEIRD population. In this way we also aim to provide an insight into how the HA functions at a multidimensional level and test the factor structure of hypothetical subdimensions of the HA. Accordingly, we hypothesized 1) Bond, Balance and Battle will be positively correlated but separate dimensions measuring the Healthy Adult mode. 2) Bond, Balance and Battle will be negatively associated with psychopathological symptoms and negative affect, and positively associated with positive affect and life satisfaction.

## Materials and methods

### Sample

Data were collected in a non-clinical community sample of 493 adult Turkish participants. The inclusion criteria comprised individuals residing in Turkey, aged between 16 and 75 years, who possessed the ability to read, write, speak, and comprehend Turkish. No additional exclusion criteria were applied beyond those implicit in the inclusion criteria. The sample consisted of 114 (24.1%) males and 358 (75.5%) females between the ages of 18 and 60 (*M* = 34.16, *SD* = 11.30).

### Materials

*Multidimensional Healthy Adult Scale (MHAS*, [15]) was developed to measure a multidimensional construct of the HA in a Turkish population. The items were created based on a prior qualitative study, which involved a thematic analysis of the HA, grounded in its original definition by Young et al. [2]. Detailed information about the qualitative analyses and factor structure can be found in our previous publication [15].

The initial item pool consisted of 80 items generated based on qualitative study [15] consistent with recommendations to start with two to three times as many items as intended for the final scale [22]. After discussion within the analysis team, 60 items were selected, with the aim of developing a questionnaire of 20–30 items. Due to time constraints, data collection had to be initiated before the thematic analysis was finalized. Therefore, a larger set of items was included in data collection to allow evaluation of which items are supported by the qualitative findings and are suitable for quantitative testing.

During the analysis of the qualitative study, thematic patterns reflecting bottom-up and top-down processing led us to retain two subscales per dimension instead of our initial expected of three. To maintain construct validity, certain items were conceptually excluded based on insights from the qualitative work conducted prior to the quantitative analyses (i.e., Items related to social support and protection/encouragement of others in Bond (11 items) and Self-Distraction in Balance (6 items), and Self-Empowerment in Battle (6 items), leaving 37 items for analysis).

Based on the qualitative analysis, a hypothesized factor structure was proposed and tested using confirmatory factor analysis. Iterative processes were employed in the SEM model by which cross loadings and poorly fitting items were deleted based on modification indices [23]. Modification indices were not used as a sole basis for model modification. We evaluated items with large modification indices in the light of theoretical relevance, content validity and redundancy using a theoretical justification. Finally, for each of three overarching constructs, Bond, Balance and Battle, six items were selected. In each scale, half of the questions represent a top-down approach while the other half represents the bottom-up approach (a comprehensive description of the top-down and bottom-up approach to the HA can be found in [15]). The final scale contained 18 items distributed across the scales Bond, Balance and Battle.

*The Brief Symptom Inventory* (BSI, [24])) is a self-report questionnaire that assesses psychopathological symptoms experienced in the last seven days. It consists of 53 items rated on a five-point Likert scale (0 = not at all, 4 = extremely). We utilized the Turkish adaptation [25] which includes five dimensions (i.e., depression, anxiety, somatization, hostility, and negative self-concept) and demonstrated acceptable reliability and validity. In the current study, the internal consistency (Cronbach alpha) of the subscales ranged between *α* =.79 and *α* =.90.

*Positive and Negative Schedule* (PANAS, [26]) is a self-report questionnaire that assesses affect. It consists of 20 items rated on 5-point Likert scales (1 = very slightly or not at all, 5 = extremely). The Turkish translation of the PANAS has demonstrated acceptable reliability and validity [27]. In the current study, the internal consistency of the subscales for both positive and negative affect was *α* = .87.

*The Satisfaction with Life Scale* (SWLS; [28]) self‐report instrument assessing the level of life satisfaction using 7‐point Likert scales (1 = strongly disagree,7 = strongly agree). The Turkish translation of the scale has acceptable reliability and validity [29] In the present study, the internal consistency of the scale was found to be *α* = 0.86.

*The Short Schema Mode Inventory* (SMI-SF) is a self-report questionnaire adapted from Lobbestael et al. [16] which is a shortened version of the original SMI [30]. It consists of 118 items rated on a 6-point Likert scale. We utilized the Turkish adaptation of the SMI-SF [31] and only employed the HA and the VC subscales. The internal consistency of the HA subscale was *α* =.76 and of the VC subscale *α* =.92.

### Procedure

We used an online survey site (Qualtrics, Provo, UT) to upload the questionnaires and created a link to the participant to fill in the questionnaires. We employed snowball sampling to recruit participants who volunteered for the study without compensation. Data were collected between September 2018, and December 2018. Participants were able to complete the questionnaires only after providing written informed consent by selecting ‘I agree’ on the first page of the Qualtrics survey. They are additionally encouraged to disseminate the study link to other potential participants in order to enhance response rates. This approach, however, should be employed with caution, as such recruitment may introduce systematic error, including volunteer self‑selection bias and deviation from random sampling procedures. Upon survey completion, participants were thanked for their participation. Approval for the study and methodology was granted by the Applied Ethics Research Center of Arel University on September 18, 2018.

### Data analysis plan

Data were analyzed using R [32] and SPSS. Lavaan (vo. 06–19; [33]) was used to do a confirmatory factor analysis (CFA) and structural equation model (SEM), in order to check the proposed factor structure as well as its content validity. Although there was very little missing data, Little’s MCAR test was used to confirm that the data was missing at random, which was confirmed. Since latent indicators were ordinal with fewer than seven response categories, they were handled as ordered variables and a DWLS estimator with robust standard errors was used. Robust test statistics are reported whenever possible [34,35].

Outliers were checked using boxplots as well as Mahalanobis distances for multivariate normality, using the influence_stat function of the Semfindr package (vo. 0.1.8 [36]) and based on the CFA model. Prior to the analyses, a Kaiser Mayer Olkin test, as well as a Bartlett test of Sphericity were done to confirm the data is fit for factor analysis. Univariate and multivariate normality were checked using skewness and kurtosis and the Henze-Zirkler test respectively. Considering the size of the sample, non-normality would not be an immediate concern due to the central limit theorem. Multicollinearity was checked between indicators as well as Maximum Likelihood estimated factor scores of the CFA, correlations above.80 would be considered concerning, VIF was also checked based on a linear model with the factor score estimates as predictors. The gender of two participants who had indicated not wanting to disclose their gender was set as missing, and binary coding was used for women (0) and men (1).

A CFA with “orthogonal” option in Lavaan was used to constrain all covariances of the latent variables, and items were classified as ordinal, and an overarching (second order) factor was estimated, consisting of the three scales. A predictive model was added, where the latent factors were used as predictors of clinical outcomes, namely psychopathological symptoms (BSI), positive affect (PANAS-P), negative affect (PANAS-N) and life satisfaction (LSI). In this model gender and age were added as control variables. Adjustments to the model (e.g., added covariances between indicators) were made depending on the modification indices. A model is considered to have sufficient fit if at least two of the following criteria are met: CFI > .90, TLI > .90, RSMEA < .08, SRMR < .08. Pearson correlations were calculated for the saved factor scores, the outcome measures and the SMI scales.

## Results

### Outliers and assumptions

Influential outliers, based on the influence test (*N* = 24) were removed from the data, leaving a sample of 450 participants. After removal of the influential outliers, there were still univariate outliers for most variables in the data, however, these were not removed due to being seen as natural variance in the data. Due to the DWLS and robust estimates, the analyses should be robust to these outliers. Both the KMO (MSA = .93) and Bartlett’s test of sphericity (*p* < .001), indicated the data was appropriate for factor analysis. We found univariate, normality, with skewness and kurtosis within range for all variables in the model, but multivariate normality was not achieved, this should not be an issue given the sample size and method of estimation. There was no multicollinearity in the data, descriptive statistics can be found in Table 1 and correlations can be found in Table 2.

**Table 1:** Descriptive statistics for the observed variables and the saved factor scores.

Variable	N	M	(SD)	min	max
Bond	450	0.00	(1.48)	−4.23	3.74
Balance	450	0.00	(2.42)	−6.93	6.79
Battle	450	0.00	(2.65)	−8.25	6.95
Healthy Adult	450	0.00	(1.00)	−2.70	3.99
Brief Symptom Inventory	445	88.22	(26.55)	53.00	193.00
Negative Affect	447	18.69	(6.72)	10.00	42.00
Positive Affect	447	32.31	(7.43)	11.00	49.00
Life Satisfaction Inventory	444	23.38	(6.75)	5.00	35.00
SMI Heathy Adult	450	47.13	(6.18)	20.00	60.00
SMI Happy Child	450	43.32	(8.10)	18.00	60.00
SMI Vulnerable Child	450	19.74	(8.43)	10.00	48.00
SMI Angry Child	450	22.85	(7.81)	9.00	48.00
SMI Punitive Parent	450	16.21	(5.63)	10.00	42.00
SMI Demanding Parent	450	34.80	(7.94)	15.00	58.00

**Table 2:** Spearman correlations for the dependent and independent variables.

Variable		2	3	4	5	6	7	8	9	10	11	12	13
1	Bond	.55	.55	−.10^b^	−.12^b^	.30	.21	.43	.47	−.17	−.09^ab^	−.11^b^	.16
2	Balance		.67	−.35	−.39	.42	.30	.61	.55	−.41	−.33	−.34	.01^ab^
3	Battle	450		−.25	−.29	.49	.31	.56	.55	−.33	−.17	−.17	.20
4	Brief Symptom Inventory	445	445		.66	−.31	−.40	−.32	−.51	.72	.65	.55	.31
5	Negative Affect	447	447	442		−.31	−.37	−.27	−.45	.58	.60	.47	.31
6	Positive Affect	447	447	442	447		.37	.43	.53	−.37	−.17	−.21	.05^ab^
7	Life Satisfaction Inventory	444	444	439	441	441		.28	.52	−.46	−.31	−.31	−.05^ab^
8	SMI Heathy Adult	450	450	445	447	447	444		.55	−.34	−.17	−.27	.20
9	SMI Happy Child	450	450	445	447	447	444	450		−.65	−.45	−.44	−.07^ab^
10	SMI Vulnerable Child	450	450	445	447	447	444	450	450		.61	.63	.31
11	SMI Angry Child	450	450	445	447	447	444	450	450	450		.56	.41
12	SMI Punitive Parent	450	450	445	447	447	444	450	450	450	450		.43
13	SMI Demanding Parent	450	450	445	447	447	444	450	450	450	450	450	

*Note.* N for each correlation is shown below the diagonal.

^a^correlations were **not** significant before correction for multiple testing.

^b^correlations were **not** significant after correcting for multiple testing. Bond, Balance and Battle correlations are based on the factor score estimates, not sum scores.

### Confirmatory factor analysis

Confirmatory factor analysis indicated an excellent fit for the measurement model (CFI = 1, TLI = .999, RSMEA = .012, SRMR = .037), with covariances between two sets of items (one in the Bond scale, and one in the Battle scale) allowed after inspection of the modification indices. Factor loadings, both unstandardized and standardized, can be found in Table 3. Standardized covariance between the two Bond items was.336, and between the two Battle items was.285. The internal consistency was acceptable to good for Bond (α = .82), Balance (α = .78) and Battle (α = .84).

**Table 3:** Factor loadings for the CFA measurement model.

Scales and items	Est.	SE	z	p	Std. Est
Bond					
1. I enjoy nurturing other people.	0.411	0.030	13.566	<.001	.609
2. I have a warm interest in other people.	0.560	0.031	17.913	<.001	.829
3. My loved ones tell me that I encourage them to achieve their goals in life.	0.474	0.029	16.121	<.001	.702
4. Other people say that I am a good listener.	0.449	0.031	14.323	<.001	.664
*5. I attune to other people’s emotions.*	0.496	0.029	17.160	<.001	.734
*6. I feel for other people.*	0.436	0.031	14.162	<.001	.645
Balance					
7. I usually have a good sense of how I am feeling.	0.321	0.04	8.025	<.001	.775
8. I pay attention to my emotions and accept them as they are.	0.285	0.036	7.923	<.001	.690
9. I know I am not able to control everything and I am at peace with that.	0.255	0.033	7.607	<.001	.615
10. I am able to stop myself when I feel that I might get hurt.	0.267	0.032	8.278	<.001	.644
11. I am good at controlling my ambitions.	0.270	0.033	8.153	<.001	.652
12. I don’t lose my temper even if I get really angry.	0.222	0.029	7.627	<.001	.536
Battle					
13. I always try to have a positive attitude.	0.286	0.045	6.338	<.001	.756
14. No matter how big the problem is, there is always hope.	0.323	0.050	6.488	<.001	.855
15. I often have the idea that everything is going to be all right in the end.	0.282	0.043	6.602	<.001	.745
*16. Challenges enhance your ability to solve problems.*	0.225	0.037	6.119	<.001	.596
*17. The challenges I have faced in life, made me realize that I actually do better than I thought.*	0.243	0.038	6.361	<.001	.642
18. *If one way does not work, I strive hard to find another even if other people give up.*	0.259	0.039	6.571	<.001	.685
Healthy Adult					
Bond	1.091	0.097	11.241	<.001	.737
Balance	2.201	0.318	6.919	<.001	.910
Battle	2.449	0.435	5.630	<.001	.926

*Note.* Sets of items within each scale in italics were allowed to covary in the tested models. Est = Estimate, Std. = standardized.

Validity and reliability of the model was tested with various measures. Average Variance Extracted (AVE) was calculated based on the final measurement model, including the higher order Healthy Adult factor and the item covariances specified previously. AVE was calculated for Bond (AVE = .491), Balance (AVE = .431), and Battle (AVE = .516). These AVE values are on the lower side, especially for Bond and Balance, and are likely related to some factor loadings being relatively low. We calculated the Composite Reliability (CR) as an additional metric of internal consistency; this showed at least acceptable consistency of Bond (CR = .829), Balance (CR = .777) and Battle (CR = .854). Heterotrait-Monotrait (HTMT) values were calculated as a measure of discriminant validity based on the Lavaan correlation table, and indicated sufficient distinctiveness between Bond and Balance (HTMT = .665), Bond and Battle (HTMT = .669) and Balance and Battle (HTMT = .836).

Measurement invariance was checked across gender. To facilitate this, two individuals that did not indicate a binary gender were removed from the dataset (N = 448). Furthermore, given the ordinal data specification, the analysis required data to be present for each ordinal category (1−6), however, eight variables had missing data for men in one of the categories, in 7 cases this was for the lowest indicator (1) in one case it was for the second lowest. For all these variables, there were relatively low instances of participants selecting 1, therefore it was decided to recategorize 1–2 for these variables. Results should be interpreted with caution, given the data tested here is not completely consistent with that of the rest of the analysis. In terms of change, a ΔCFI ≤ −.01 and ΔRMSEA ≤ .015, and ΔSRMR ≤ .03 for metric and ≤.01 for scalar and strict invariance would be considered acceptable [37]. CFI, RSMEA and SRMR information and model comparison can be found in Table 4. Although usually only CFI and RSMEA are included, SRMR was included as this may be more reliable for ordinal data [38]. Model fit indices remain good across measurement invariance testing. The change metrics meet some but not all, of the ideal cut-off values: i.e., metric invariance across gender was not supported. Additional metrics are reported in Table 4. These results may also have been affected by unequal sample size, meaning they should be interpreted with care.

**Table 4:** Measurement invariance across gender.

Type	CFI	RSMEA	SRMR	ΔCFI	ΔRSMEA	ΔSRMR
Configural	1.000	.000	.046			
Metric	.997	.027	.051	−.003	.027	.006
Scalar	.999	.017	.047	.002	−.011	−.005
Strict	.999	.017	.047	.000	.000	.000

*Note.* Δ indicates change in the metric compared to the previous level of invariance. Adjustments made to facilitate this check can be found in the text.

### Predictive model

**Fig 1:**
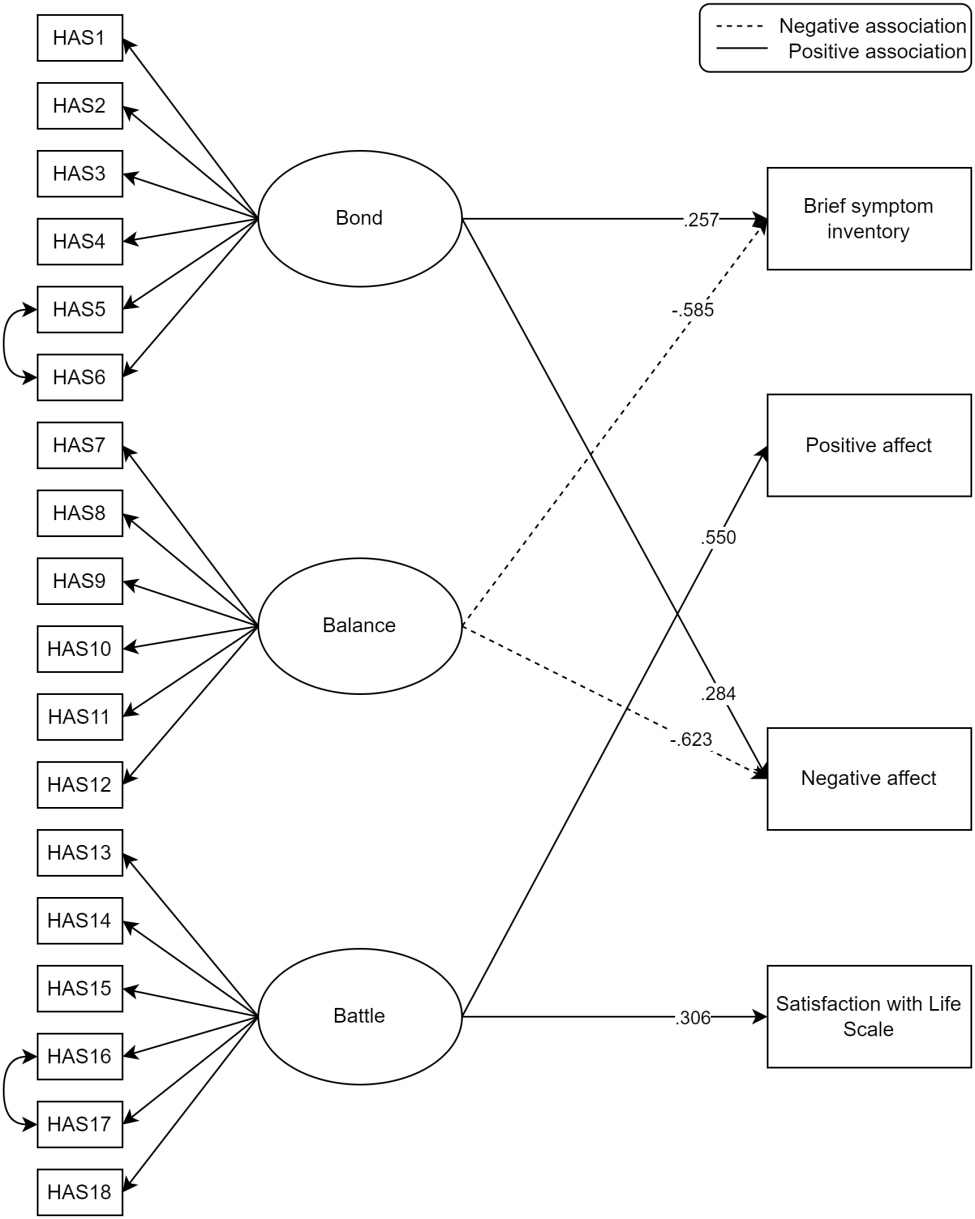
SEM model with standardized regression coefficients for outcome variable. Only significant associations are displayed, represented by arrows.

Regression results for simple SEM models (with one of the scales predicting each outcome variable, plus gender and age as control variables) can be found in Table 5. The simple models found each scale to significantly predict each of the outcomes in the expected directions. The full structural equation model used 436 observations and included Bond, Balance and Battle as predictors of BSI, Positive and Negative Affect and LSI (with gender and age as control variables). The model showed excellent fit (CFI = .990, TLI = .990, RSMEA = .041, SRMR = .039) and regression results are shown in Table 5, a visual overview of the model can be found in Fig 1. For both BSI and Negative Affect, Balance was a significant negative predictor as expected, however, Bond was a significant positive predictor. For both Positive Affect and LSI, Battle was a significant positive predictor, in line with expectations. Given the results we checked a moderation model using double mean centering in Lavaan, with Bond as an independent variable and Balance as a moderator (and gender and age as control variables). None of the interaction effects were significant, for BSI (*p* = .233), Negative Affect (*p =* .839), Positive Affect (*p* = .984) or LSI (*p* = .180).

**Table 5:** Regression coefficients for univariate regressions with each scale.

		b	SE	z	p	β
Bond	Brief Symptom Inventory	−4.172	0.764	−5.461	<.001	−.307
	Positive Affect	1.921	0.243	7.9	<.001	.508
	Negative Affect	−1.031	0.194	−5.311	<.001	−.300
	Life Satisfaction Inventory	1.319	0.203	6.481	<.001	.390
Balance	Brief Symptom Inventory	−4.022	0.653	−6.159	<.001	−.328
	Positive Affect	1.785	0.232	7.689	<.001	.523
	Negative Affect	−1.006	0.162	−6.199	<.001	−.325
	Life Satisfaction Inventory	1.24	0.195	6.36	<.001	.407
Battle	Brief Symptom Inventory	−2.911	0.656	−4.437	<.001	−.313
	Positive Affect	1.328	0.256	5.193	<.001	.513
	Negative Affect	−0.724	0.166	−4.366	<.001	−.308
	Life Satisfaction Inventory	0.915	0.193	4.752	<.001	.396

Note. Gender and age were controlled for, all models were based on 436 observations.

## Discussion

This study aimed to test the factor structure and validation of Multidimensional Healthy Adult Scale, an assessment tool that measures the HA as a multidimensional construct based on the definition of the HA [2]. We created separate subscales with empathy and providing compassion to others (Bond), Self-reflection and emotion regulation (Balance), and the individuals capacity to fight back against hardship (Battle). The items of the scale were developed based on a qualitative study conducted with people who have relatively high HA mode [15]. The hypothesized subdimensions of the HA is tested and the measurement model including Bond, Balance and Battle factors, as well as an overarching HA, demonstrated an excellent fit to the data, supporting a three-factor model. The predictive model further indicated distinct relationships between the three sub-scales and the relevant outcomes. More specifically, those who scored low on Balance are more likely to experience psychopathological symptoms and negative affect, while those who scored high on Battle, were more likely to be more satisfied with life and experience positive affect. These results aligned with expectations, further highlighting the distinct impact of the two subscales on positive and negative affective states, as well as their broader implications.

Although the measurement model showed an excellent fit, not all factor loadings were very high. This may have contributed to the relatively lower AVE values (especially for Balance), despite both fit and internal consistency being adequate to good. Moreover, although measurement invariance testing showed some tendency towards invariance between men and women, the skewed sample distribution makes these findings relatively unreliable. Revision of the measurement model might be in order, and further results should be interpreted with care. Discriminant validity between the factors was confirmed.

An unexpected finding was that, when controlled for Balance and Battle, Bond was a positive predictor for both psychopathological symptoms and negative affect, meaning that individuals who are more empathic and compassionate towards others are more likely to experience psychopathological symptoms and negative affect. However, the uncontrolled association between Bond and psychopathological symptoms and negative affect was negative, as expected. Thus, the sign of the associations reversed only after Balance and Battle were included into the model. This is a manifestation of Simpson’s paradox, where the relationship reverses direction when other variables are included in an equation; this phenomenon has been reported to occur more often than is typically acknowledged in psychological research [39]. It is important to consider the context of Bond and Balance as separate but related constructs, however, it is essential to discuss the specific relation between Bond and Balance to understand this reversal, as Battle was not found to be a predictor of negative effect or psychopathological symptoms in the univariate regression.

Bond reflects the side of the HA which is mainly associated with taking care of the needs of the VC which is related to compassion, empathy and emotional attunement [15]. Empathy and compassion are known to be linked to both positive [40,41] and negative [42–44] mental health outcomes, the latter often referred to as ‘compassion stress injury’ or ‘empathy fatigue’ [45,46]. More specifically, studies show that increased other-directed compassion [42] and affective empathy (emotional state-matching) were linked to adverse mental health outcomes, while cognitive empathy (perspective-taking) and self-compassion were not [45]. However, de Waal [47] criticizes psychologist to often neglect affective empathy: “…Without emotional engagement induced by state-matching, perspective-taking would be a cold phenomenon that could just as easily lead to torture as to helping” (p.287). Similarly, emotional attunement to the VC is a very important part for especially bottom-up approach of the HA. Thus, our conceptualization of the Bond as a dimension of the HA underlines the dual dimensions of empathy (affective vs. cognitive) and compassion (i.e., self and other oriented) because both are needed for good (re)parenting, while highlighting a potential mental health burden that may accompany heightened emotional attunement and compassion toward others. Interestingly, this burden is only visible when Balance is in the equation. It could be argued that Balance, as the most influential subscale in our study, likely accounts for variance in Bond that relates to cognitive empathy and compassion towards self. Consequently, the additional variance that Bond explains, beyond what Balance accounts for, may primarily reflect affective empathy and compassion towards others that might be associated with negative affect and psychological symptoms.

Balance involves setting limits, self-reflection and accepting of emotions. It can be more related to factors such as compassion towards the self via limit setting, accepting emotions and cognitive emotion regulation. Research consistently shows a strong negative correlation between emotion regulation and psychopathological symptoms [48], supporting our findings. We found that Balance had the highest correlation with the original HA Subscale of the Schema Mode Inventory which was found to be a central mechanism of change [10]. Studies highlight the significant role of emotion regulation in shaping the link between empathy and psychopathological symptoms. Cho and Lee [45] found that individuals with higher emotion regulation capacities experienced less compassion fatigue. Similarly, Tully et al. [49] found that reacting to others’ struggles with moderate levels of both affective and cognitive empathy, when combined with good emotion regulation was the most adaptive in terms of depressive symptoms. In the current study, the interaction term between Bond and Balance was not significant. Nonetheless, understanding how Bond and Balance interact remains an important question for future research, as the present study may lack sufficient statistical power to detect smaller or more nuanced effects.

Another finding is that both Balance and Battle contribute uniquely to healthy functioning. Battle was the only factor relating to positive affect and life satisfaction. This factor encompasses hope and self-empowerment, embracing challenges which promotes resilience. Positive emotions are demonstrated as stronger predictors of building resilience and increasing life satisfaction and these advantages can persist even when people experienced negative emotions [50]. Therefore, our finding overlaps with the recent efforts in schema therapy to argue that for a full functioning HA, the absence of symptoms is not enough, but the presence of strengths promoting life satisfaction, positive emotions and self-efficacy, are also important [17,51]. Overall, while the Balance factor emerged as the most influential predictor, Battle appears to have a stronger impact when specifically predicting positive emotions and life satisfaction.

Our findings underline the need for a better understanding of the multidimensional structure of the HA. MHAS was developed and tested in Türkiye and the items were developed based on the qualitative study rather than solely on a theory-driven method. MHAS is the first of its kind in schema therapy, being based on data from a non-Western culture and emphasizing the importance of connectedness to others and resilience. Therefore, it is still important to test the factor structure in culturally diverse and clinical populations. Empirical testing of the complex relationship between Bond and Balance is also important in a cultural context. Affective empathy is found to be moderately correlated to clinical anxiety in the data derived from Middle East and East Asia in contrast to data derived from North America and Europe where the correlation was small [43]. Thus, culture might influence the display of the HA. In addition, we only tested linear relationships between the subscales of the HA and the related outcome variables. There is empirical evidence that the relation between empathy and depression is quadratic [49]. Thus, it is also important to test for non-linear relations between variables.

Still, this study has several limitations to consider. First, because our sample is non-clinical, we cannot generalize the findings to clinical populations. Second, the cross-sectional data restricts our understanding of the relationship between HA dimensions and prevents causal conclusions. Third, while data were collected in Türkiye, the sample was obtained via snowball sampling in Istanbul, which culturally aligns more closely to individualistic cultures and lack diversity, which limits generalizability. It should also be noted that the sample is characterized by a predominance of female participants and individuals with higher levels of education, which limits its representativeness of the general population in Türkiye. Similarly, conclusions regarding structural stability across genders should be interpreted with caution. In addition, the use of snowball sampling may have introduced self-selection bias, further limiting the representativeness of the sample. Furthermore, although the literature over the gender differences in empathy and compassion is robust [41,46] our sample predominantly consists of female participants. Due to time constraints, only a limited amount of questionnaires were included in the current study, limiting what can be said about construct validity to those constructs measured.

Despite these limitations, we do believe that these early results show that there is merit in measuring the HA as a multidimensional rather than unidimensional construct. We recognize that revision of the questionnaire (either in the wording of the specific questions or the questions used) might be needed. We would also recommend future studies use more instruments that can also indicate proper discriminant validity of the subscales, including instruments measuring resilience and self-compassion. Replication of these findings in clinical populations, diverse cultural contexts, and through the application of time series models might deliver more reliable results for the use of the MHAS.

## Conclusions

To conclude, we developed a new scale to measure the HA based on a qualitative study with utilizing a bottom-up approach, tested its factor structure and association with various indices of mental well-being. The measurement model including Balance, Bond and Battle factors demonstrated an excellent fit to the data, supporting a three-factor model. The Balance dimension was associated with psychopathological symptoms and negative affect negatively, while Battle is the only factor relating to positive affect and life satisfaction. Although Bond demonstrates a positive relationship wellbeing, and with Balance and Battle, a high score on Bond, when Balance and Battle are taken into consideration, may in fact be associated with an increase in negative affect and psychopathological symptoms, which may be an example of Simpson’s paradox.

## References

[1] Young JE. Cognitive therapy for personality disorders: A schema-focused approach. Sarasota (FL): Professional Resource Press; 1999.

[2] Young JE, Klosko JS, Weishaar ME. Schema therapy: A practitioner’s guide. New York: Guilford Press; 2003.

[3] Joshua PR, Lewis V, Kelty SF, Boer DP. Is schema therapy effective for adults with eating disorders? A systematic review into the evidence. Cogn Behav Ther. 2023;52(3):213–31. 10.1080/16506073.2022.2158926 36633136

[4] Kopf-Beck J, Müller CL, Tamm J, Fietz J, Rek N, Just L, et al. Effectiveness of schema therapy versus cognitive behavioral therapy versus supportive therapy for depression in inpatient and day clinic settings: A randomized clinical trial. Psychother Psychosom. 2024;93(1):24–35. 10.1159/000535492 38176391 PMC10880804

[5] Peeters N, van Passel B, Krans J. The effectiveness of schema therapy for patients with anxiety disorders, OCD, or PTSD: A systematic review and research agenda. Br J Clin Psychol. 2022;61(3):579–97. doi: 10.1111/bjc.12324 34296767 PMC9544733

[6] Arntz A, Jacob GA, Lee CW, Brand-de Wilde OM, Fassbinder E, Harper RP, et al. Effectiveness of predominantly group schema therapy and combined individual and group schema therapy for borderline personality disorder: A randomized clinical trial. JAMA Psychiatry. 2022;79(4):287–99. doi: 10.1001/jamapsychiatry.2022.0010 35234828 PMC8892362

[7] Bamelis L, Evers SMAA, Spinhoven P, Arntz A. Results of a multicenter randomized controlled trial of the clinical effectiveness of schema therapy for personality disorders. Am J Psychiatry. 2014;171(3):305–22. 10.1176/appi.ajp.2013.12040518 24322378

[8] Giesen-Bloo J, Van Dyck R, Spinhoven P, Van Tilburg W, Dirksen C, Van Asselt T. Outpatient psychotherapy for borderline personality disorder: Randomized trial of schema-focused therapy vs transference-focused psychotherapy. Arch Gen Psychiatry. 2006;63(6):649–58. 10.1001/archpsyc.63.6.649 16754838

[9] Arntz A, Jacob G. Schema therapy in practice: An introductory guide to the schema mode approach. Chichester: Wiley-Blackwell; 2013.

[10] Yakın D, Grasman R, Arntz A. Schema modes as a common mechanism of change in personality pathology and functioning: Results from a randomized controlled trial. Behav Res Ther. 2020;126:103553. doi: 10.1016/j.brat.2020.103553 32018065

[11] Fassbinder E, Schweiger U, Martius D, Brand-de Wilde O, Arntz A. Emotion regulation in schema therapy and dialectical behavior therapy. Front Psychol. 2016;7:1373. 10.3389/fpsyg.2016.01373 PMC5021701 27683567

[12] Lockwood G, Perris P. A New Look at Core Emotional Needs. The Wiley‐Blackwell Handbook of Schema Therapy. Chichester: Wiley; 2012. p. 41–66. doi: 10.1002/9781119962830.ch3

[13] Lockwood G, Samson R. Understanding and meeting core emotional needs. Creative methods in schema therapy: Advances and innovation in clinical practice. New York: Routledge/Taylor & Francis Group; 2020. p. 76–90.

[14] Aalbers G, Engels T, Haslbeck JMB, Borsboom D, Arntz A. The network structure of schema modes. Clin Psychol Psychother. 2021;28(5):1065–78. 10.1002/cpp.2577 33606318 PMC8596577

[15] Yakın D, Arntz A. Understanding the reparative effects of schema modes: an in-depth analysis of the healthy adult mode. Front Psychiatry. 2023;14:1204177. doi: 10.3389/fpsyt.2023.1204177 37941965 PMC10628052

[16] Lobbestael J, van Vreeswijk M, Spinhoven P, Schouten E, Arntz A. Reliability and validity of the short Schema Mode Inventory (SMI). Behav Cogn Psychother. 2010;38(4):437–58. doi: 10.1017/S1352465810000226 20487590

[17] Louis JP, Wood AM, Lockwood G, Ho MHR, Ferguson E. Positive clinical psychology and schema therapy (ST): The development of the Young Positive Schema Questionnaire (YPSQ) to complement the Young Schema Questionnaire 3 Short Form (YSQ-S3). Psychol Assess. 2018;30(9):1199–213. 10.1037/pas0000567 29672073

[18] Louis JP, Wood AM, Lockwood G. Development and validation of the positive parenting schema inventory (PPSI) to complement the young parenting inventory (YPI) for schema therapy (ST). Assessment. 2020;27(4):766–86. 10.1177/1073191118798464 30193528

[19] Pilkington PD, Younan R, Karantzas GC. Identifying the research priorities for schema therapy: A Delphi consensus study. Clin Psychol Psychother. 2023;30(2):344–56. doi: 10.1002/cpp.2800 36369615

[20] Henrich J, Heine SJ, Norenzayan A. The weirdest people in the world?. Behav Brain Sci. 2010;33(2–3):61–83. 10.1017/S0140525X0999152X 20550733

[21] Kağitçibaşi Ç. Family, self, and human development across cultures: theory and applications. New York: Taylor & Francis; 2017.

[22] Boateng GO, Neilands TB, Frongillo EA, Melgar-Quiñonez HR, Young SL. Best practices for developing and validating scales for health, social, and behavioral research: A primer. Front Public Health. 2018;6:0. doi: 10.3389/fpubh.2018.00100 PMC6004510 29942800

[23] Reise SP, Waller NG, Comrey AL. Factor analysis and scale revision. Psychol Assess. 2000;12(3):287–97. doi: 10.1037//1040-3590.12.3.287 11021152

[24] Derogatis LR. The Brief symptom inventory: An introductory report. Psychol Med. 1983;13(3):595–605. 6622612

[25] Şahin NH, Durak A. Adaptation of the brief symptom inventory for the Turkish youth. Turkish Journal of Psychology. 1994;9(31):44–56.

[26] Watson D, Clark LA, Tellegen A. Development and validation of brief measures of positive and negative affect: The PANAS scales. J Pers Soc Psychol. 1988;54(6):1063–70. doi: 10.1037//0022-3514.54.6.1063 3397865

[27] Gençöz T. Pozitif ve Negatif Duygu Ölçeği: Geçerlik ve güvenirlik çalışması. Türk Psikoloji Dergisi. 2000;15(46):19–26.

[28] Diener E, Emmons RA, Larsen RJ, Griffin S. The satisfaction with life scale. J Pers Assess. 1985;49(1):71–5. doi: 10.1207/s15327752jpa4901_13 16367493

[29] Durak M, Senol-Durak E, Gencoz T. Psychometric properties of the satisfaction with life scale among Turkish university students, correctional officers, and elderly adults. Soc Indic Res. 2010;99(3):413–29. 10.1007/s11205-010-9589-4

[30] Young JE, Arntz A, Atkinson T, Lobbestael J, Weishaar M, Vreeswijk MF, et al. The Schema Mode Inventory. New York: Schema Therapy Institute; 2007.

[31] Aytaç M, Köse Karaca B, Karaosmanoğlu A. Turkish adaptation of the Short Schema Mode Inventory. Clin Psychol Psychother. 2020;27(3):346–63. doi: 10.1002/cpp.2432 31999383

[32] Core R, Team. R: The R Project for Statistical Computing. https://www.r-project.org/. 2024. Accessed 2025 February 23.

[33] Rosseel Y. lavaan: An R package for structural equation modeling. J Stat Softw. 2012;48:1–36.

[34] Rhemtulla M, Brosseau-Liard PÉ, Savalei V. When can categorical variables be treated as continuous? A comparison of robust continuous and categorical SEM estimation methods under suboptimal conditions. Psychol Methods. 2012;17(3):354–73. doi: 10.1037/a0029315 22799625

[35] Li C-H. The performance of ML, DWLS, and ULS estimation with robust corrections in structural equation models with ordinal variables. Psychol Methods. 2016;21(3):369–87. doi: 10.1037/met0000093 27571021

[36] Cheung SLM. Package “semfindr” title influential cases in structural equation modeling. https://CRAN.R-project.org/package=semfindr. 2024. Accessed 2025 February 23.

[37] Chen FF. Sensitivity of goodness of fit indexes to lack of measurement invariance. Structural Equation Modeling: A Multidisciplinary Journal. 2007;14(3):464–504. doi: 10.1080/10705510701301834

[38] Shi D, Maydeu-Olivares A, Rosseel Y. Assessing fit in ordinal factor analysis models: SRMR vs. RMSEA. Structural Equation Modeling: A Multidisciplinary Journal. 2019;27(1):1–15. doi: 10.1080/10705511.2019.1611434

[39] Kievit RA, Frankenhuis WE, Waldorp LJ, Borsboom D. Simpson’s paradox in psychological science: A practical guide. Frontiers in Psychology. 2013;4(AUG):54928. doi: 10.3389/fpsyg.2013.05428 PMC3740239 23964259

[40] Yin Y, Wang Y. Is empathy associated with more prosocial behaviour? A meta‐analysis. Asian J of Social Psycho. 2022;26(1):3–22. doi: 10.1111/ajsp.12537

[41] Lee EE, Govind T, Ramsey M, Wu TC, Daly R, Liu J, et al. Compassion toward others and self-compassion predict mental and physical well-being: A 5-year longitudinal study of 1090 community-dwelling adults across the lifespan. Transl Psychiatry. 2021;11(1):397. doi: 10.1038/s41398-021-01491-8 34282145 PMC8287292

[42] López A, Sanderman R, Ranchor AV, Schroevers MJ. Compassion for others and self-compassion: Levels, correlates, and relationship with psychological well-being. Mindfulness (N Y). 2018;9(1):325–31. 10.1007/s12671-017-0777-z 29387268 PMC5770484

[43] Nair TK, Waslin SM, Rodrigues GA, Datta S, Moore MT, Brumariu LE. A meta-analytic review of the relations between anxiety and empathy. J Anxiety Disord. 2024;101:102795. 10.1016/j.janxdis.2023.102795 38039916

[44] Yan Z, Zeng X, Su J, Zhang X. The dark side of empathy: Meta-analysis evidence of the relationship between empathy and depression. Psych J. 2021;10(5):794–804. doi: 10.1002/pchj.482 34494388

[45] Cho H, Lee DG. Effects of affective and cognitive empathy on compassion fatigue: Mediated moderation effects of emotion regulation capability. Pers Individ Dif. 2023;211:112264. doi: 10.1016/j.paid.2023.112264

[46] Russel M, Brickel M. The “double-edge sword” of human empathy: A unifying neurobehavioral theory of compassion stress injury. Social Sciences. 2015;4(4):1087–117. 10.3390/socsci4041087

[47] de Waal FBM. Putting the altruism back into altruism: the evolution of empathy. Annu Rev Psychol. 2008;59:279–300. doi: 10.1146/annurev.psych.59.103006.093625 17550343

[48] Lincoln TM, Schulze L, Renneberg B. The role of emotion regulation in the characterization, development and treatment of psychopathology. Nat Rev Psychol. 2022;1(5):272–86. doi: 10.1038/s44159-022-00040-4

[49] Tully EC, Ames AM, Garcia SE, Donohue MR. Quadratic associations between empathy and depression as moderated by emotion dysregulation. J Psychol. 2016;150(1):15–35. doi: 10.1080/00223980.2014.992382 25565484

[50] Cohn MA, Fredrickson BL, Brown SL, Mikels JA, Conway AM. Happiness unpacked: Positive emotions increase life satisfaction by building resilience. Emotion. 2009;9(3):361. 10.1037/a0015952 19485613 PMC3126102

[51] Miklósi M, Vajsz K, Oláh S, Nagy V, Szabó B. An investigation of the Bernstein’s strengths Scale: Factorial validity and network analysis of attention-deficit/hyperactivity symptoms, mental health, and the strengths of the healthy adult self. BMC Psychiatry. 2024;24(1):725. doi: 10.1186/s12888-024-06156-6 39443890 PMC11515520

